# Exoscopic versus Microscopic Surgery in 5-ALA-Guided Resection of High-Grade Gliomas

**DOI:** 10.3390/jcm13123493

**Published:** 2024-06-14

**Authors:** Giada Garufi, Alfredo Conti, Bipin Chaurasia, Salvatore Massimiliano Cardali

**Affiliations:** 1 Department of Neurosurgery, Azienda Ospedaliera Papardo, University of Messina, 98158 Messina, Italy; scardali@unime.it; 2 Department of Neurosurgery, IRCCS Istituto delle Scienze Neurologiche di Bologna, 40139 Bologna, Italy; alfredo.conti2@unibo.it; 3 Dipartimento di Scienze Biomediche e Neuromotorie (DIBINEM), Alma Mater Studiorum Università di Bologna, Via Altura 3, 40123 Bologna, Italy; 4 Department of Neurosurgery, Neurosurgery Clinic, Birgunj 44300, Nepal; trozexa@gmail.com; 5 Department of Biomedical, Dental and Morphological and Functional Imaging, University of Messina, Via Consolare Valeria, 98125 Messina, Italy

**Keywords:** exoscope, microscope, 5-ALA, high-grade glioma

## Abstract

**Background:** Glioma surgery has been remarkably enhanced in the past 2 decades, with improved safety and limited but improved life expectations. The fluorescence-guided resection of high-grade gliomas (HGGs) plays a central role in this sense, allowing a greater extent of resection (EOR). The introduction of exoscopic-guided surgery may be considered in implementing fluorescence techniques over traditional microscopes. We present the application and the advantages of exoscopic-guided surgery compared to microscopic surgery in tumor resection guided by 5-ALA fluorescence in patients with HGGs. **Methods:** Ten consecutive patients underwent surgery for HGG resection. The surgery was performed via an exoscopic-guided procedure (Olympus ORBEYE) and after the oral administration of Gliolan 5 h before the procedure. During surgery, the procedure shifted to using a microscopic (Kinevo 900, Zeiss) view. The intensity of the fluorescence under the two different procedures was subjectively measured in different picture samples during the surgery on a 1 to 5 (from minimum to maximum) scale. The brightness of the surgical field and the detailing of the anatomy were also analyzed comparatively. **Results:** Among the ten patients, the histopathological diagnosis was an high-grade glioma in all cases. In nine cases, it was possible to achieve gross total resection. There was no perioperative mortality. The median fluorescence intensity, on a scale of 1–5, was 4.5 in the exoscope group and 3.5 in the microscope group (*p* < 0.01). **Conclusions:** The exoscopic-guided surgery adds advantages to traditional fluorescence-guided surgery with 5-aminolevulinic acid. Beyond the important advantage of low cost and the possibility to perform collaborative surgeries, it adds a plain and continuous visualization of the tumor and offers advantages in the surgical field of fluorescence-guided glioma surgery compared to the microscopic-guided one.

## 1. Introduction

Gliomas are aggressive tumors of the brain which account for about 80% of all primary malignant cerebral tumors [1,2]. For their aggressive behavior, they require multimodal management: the current treatment of choice is extensive surgical resection, accompanied by chemotherapy and radiotherapy. When achievable, a gross total resection (GTR) up to 98% is the first step and it is known to be correlated with a significant survival advantage [3,4]. However, the prognosis of high-grade gliomas (HGGs) remains poor because of their infiltrative nature and the fast local relapse rate. The literature reports that more than 80% of HGG recurrences occur within 2 cm of the resection margins [5]. New technologies including neuronavigation intraoperative ultrasound, intraoperative neurophysiological monitoring (IONM), exoscopic guidance and fluorescence techniques have been introduced in the last few decades [6]. The benefits of fluorescence-guided surgery, particularly with 5-aminolevulinic acid (5-ALA) in the resection of HGGs have been documented in several studies [7]. The U.S. Food and Drug Administration (FDA) approved 5-ALA (Gleolan^®®^; Photonamic GmbH & Co. KG, Pinneberg, Germany) for use as an intraoperative optical imaging agent in patients with suspected high-grade gliomas in 2017. The approval occurred a decade after European approval and a multicenter, phase III randomized trial which confirmed that surgeons using 5-ALA as a surgical adjunct could achieve more complete resections of tumors in HGG patients and better patient outcomes than with conventional microsurgery [1]. 5-ALA is a well-tolerated fluorophore with low rates of adverse events, the most common of them involving liver metabolism, temporary hypotension and light sensitivity for the first 24 h after application. Compared to other fluorophores, as well as sodium fluorescein (SF), 5-ALA has proven to have more sensitivity and sensibility in detecting primary tumor cells thanks to the peculiar accumulation of metabolically active glioma cells in contrast to surrounding healthy brain parenchyma [8,9]. After oral administration, 5-ALA passes the blood–brain barrier (BBB) and it is converted into the auto fluorescent marker protoporphyrin IX (PpIX), acting like a marker of cancerous cells. Until now, a proper visualization of PPIX fluorescence required the blue filters of traditional xenon microscopes, a blue-violet light with a wavelength of 375 to 440 nm and an emission filter, thus allowing the visualization of pink-red fluorescence, with an emission peak at 635 and 704 nm. Exoscopic-guided surgery, thanks to the outer 3D 4K screen and the LED light source, combined with a blue filter could be considered a favorable adjunct compared to the traditional microscopic-guided surgery. The ORBEYE 3D exoscope system was the first to be enriched with 4K HD technology and concurrently 3D visualization, thus providing a clear and detailed surgical field. The operating system for the exoscope is Unix-like and it is controlled by a graphical user interface established within the console itself. The hardware is composed of two metal-oxide semiconductor cameras, with a resolution of 3840 × 2160 pixels. The camera has an individual counter-balance and the arm body itself contains a dead man’s switch and the controls for zoom and focus. The light source of the ORBEYE exoscope is carried out by a fiber-optic LED source, different to the xenon light of the microscope. The image is created through a 3D line-by-line (LBL) mode that can be displayed in outer 3D 4K monitors that use the same resolution as the capturing cameras: to proper visualize the final image, the surgeon is provided with circularly polarized 3D glasses. The magnification ranges between 1.1 and 25.8 times, using a zooming ratio of 1:12 times. The focal length is 220–550 mm and focus with a field of view ranges from 7.5 to 171 mm. The exoscope is supplied with light filters for 5-aminolevulinic acid and indocyanine video angiography [10]. The advantages of using an exoscope is the visualization of the surgical field in an outer 3D 4K display, which generally has either an equivalent or higher resolution than that of an OM [11,12,13]. The LED light source gives other notable adjuncts to neurosurgery because it generates less heat than the halogen bulbs used in an operative microscope, avoiding thermal injury to tissues within the surgical field. The LED light source is reported to obtain a more accurate and color-contrasted surgical field: this could be potentially beneficial for the surgeon in order to easily recognize anatomical details, vessels or bleedings both in the white- and the blue-filtered outer pictures [12,14]. In this report, we describe our preliminary experience in the 5-ALA-guided HGG surgery adopting exoscopic guidance (Olympus ORBEYE) as an alternative or complement to traditional microscope-guided surgery in order to achieve a better visualization of the surgical field and to obtain the highest degree of tumor resection.

## 2. Materials and Methods

Ten consecutive patients underwent surgery for the resection of an intra-axial lesion from February 2022 to February 2023. These procedures were carried out following normal clinical practice at Azienda Ospedaliera Papardo Messina, Sicily. This study did not require ethical committee approval: all patients were fully informed about the procedure and they gave their written consent for both the procedure and for enrolling in this study. Clinical and personal data were kept confidential. An objective analysis of the features selected in this paper were carried out through Python (Phyton 3.12.4 software foundation).

### 2.1. Statistical Analysis

A descriptive analysis and parametric tests were used (SPSS Statistics v29). The level of statistical significance was set at *p* < 0.01.

### 2.2. Preoperative Procedures

All patients, 6 males and 4 females (Table 1), underwent preoperative MRI + gadolinium to assess the tumor volume and the tumor localization. The StealthStation^®^ surgical navigation system (Medtronic, Inc., Minneapolis, MI, USA) was used for preoperative and intraoperative neuronavigation in all cases. Neurophysiological intraoperative monitoring by transcranial or cortical stimulation for motor-, visual- and somatosensorial-evoked potentials was performed in all cases. Antibiotic prophylaxis was performed in all patients through a protocol of 2 g of cephalosporin 2 h before surgery.

### 2.3. 5-ALA Administration

5-ALA (Gleolan^®^; Photonamic GmbH & Co. KG) is supplied to the patient 3–5 h before the procedure. A dose of 20 mg/kg of Gleolan is administered orally to the patient in 50 mL of drinking water. Then, the patient is given ALA 3 h before surgery and is protected from strong light exposure within the first 24 h after 5-ALA administration to avoid risks related to photosensitivity.

### 2.4. Exoscopic 5-ALA-Guided Surgery Method

The surgery was performed thanks to the ORBEYE exoscope (OE) (Olympus) which has a camera with an LED light source, an ergonomic arm and an outer 3D, 4K 3D 55 screen which allows a natural depth of field visualization with a high resolution of anatomical details. The blue light excitation (λ = 400–410 nm) allows one to detect protoporphyrin IX, the final 5-ALA metabolite accumulated in the tumor cells, which is strongly fluorescent (peak λ = 635 nm). Fluorescence emission was classified as intense (solid or lava-like) red fluorescence, corresponding to tumor tissue (vital and solid), and faint pink or pinkish fluorescence, corresponding to infiltrating tumor cells. Normal tissue not accumulating protoporphyrin IX reflects the blue-violet light, which appeared blue and was classified as blue [15] (Table 2).

Before the surgery, a traditional operative microscope (OM) (Kinevo i900, ZEISS, Oberkochen, Germany) with a blue filter was used during the procedure, just for illustrative and comparative use. The surgery consisted of a gross total resection in 9 patient and in a subtotal resection in 1 patient. Resection was achieved by alternating white and blue light to complete exeresis. The intensity of the fluorescence was subjectively measured in different picture samples during the surgery on a 1 to 5 (from minimum to maximum) scale. The same frames were analyzed with the microscope and graded on a 1 to 5 scale. The brightness of the surgical field and the detailing of the anatomy were also analyzed comparatively (Table 3). Finally, we provided an objective analysis of different features of both scopes in terms of exposure, fluorescence intensity and brightness.

In order to ensure the same picture frames were analyzed with both the OM and OE, we took into consideration the following factors: 1. Hemostasis: Pictures were taken after the hemostasis was reached. If any bleeding occurred while the two pictures were taken, the surgeon needed to reach hemostasis. Then, the pictures were taken again both with the exoscope and the microscope in order to compare the exact surgical field without bleedings. 2. The modality of the collection of pictures with the OM and OE: when the surgeon found a good intraoperative view, thus representative for our objective, the first picture was taken with the OE; then, the exoscope was removed, the OM was brought in and the second picture was taken with no intervening resection.

An MRI scan was performed in all of the craniotomy cases within the first 72 postoperative hours to assess the extent of resection.

## 3. Results

In our study, 10 patients, 4 female and 6 male, underwent surgery for the exeresis of a high-grade glioma; the age range was 39 to 80 years, with a median of 62.1 (interquartile range = 54–71). No complication or adverse reactions (5-ALA-related) were observed, except for a case of a slight increase in serum GGT and a case of photosensibilization, but both cases were resolved straight away. There was no perioperative mortality. The median fluorescence intensity, according to the subjective assessment of the surgeons on a scale of 1–5, was 4.7 in the OE group (IQR = 4.5–5) and 4.0 (IQR 4–4.125) in the OM group; the brightness of the surgical field was evaluated as 4.9 (IR 4.9–5) in the OE group and 3.85 in the OM group (IR 3.5–4); the detailing of the anatomy with the blue filter on the OE was evaluated with a median of 4.5 (IQR 4–5) in the OE group and 3.2 (IQR 3–3.5) in the OM group. According to the comparative analysis, there was statistical significance between the two groups (*p* < 0.01) (Table 4).

Finally, we provided an objective analysis of different features of both scopes in terms of fluorescence intensity and brightness. The comparative analysis was made in the same pictures evaluated subjectively from the surgeons in terms of fluorescence intensity and brightness. These features were analyzed separately in three different areas of the surgical field (red, pink and blue). The median fluorescence intensity, according to the objective assessment, was 230–255 in the red areas, 170–210 in the pink areas and 80–140 in the blue areas in the OM group (range 80–255) and 220–250 in the red areas, 190–220 in the pinkish areas and 100–180 in the blue areas in the OE group (range 100–250) (*p* > 0.01). Regarding the brightness (median) of the field, we analyzed comparative pictures through the RGB (red, green and blue scale) with brightness values from 0 to 255, where 0 was completely black and 255 was maximum brightness. The red areas resulted in R = 255, G = 50 and B = 50 values; the pink areas resulted in an R = 235, G = 130 and B = 200; the blue areas resulted in an R = 80, G = 120 and B = 255 in the OM group, with a range of 50–255; the red area resulted in a brightness range of R: 255, G: 120 and B: 90; the pink areas resulted in brightness values of R: 245, G: 200 and B: 110; and the blue area resulted in brightness values of R: 140, G: 170 and B: 210, with a range of 80–255 (*p* < 0.01) (Table 5 and Table 6). According to the comparative analysis, there was statistical significance between the two groups (*p* < 0.01).

## 4. Discussion

**Figure 1:**
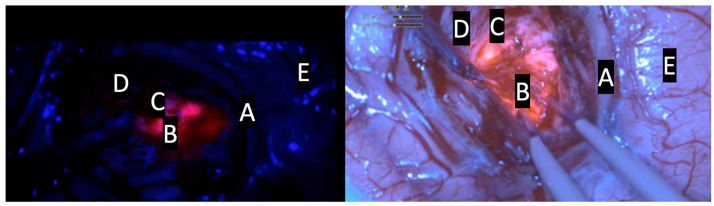
Comparative intraoperative pictures of patient N.9; kinevoi900 (**left side**); Olympus ORBEYE exoscope (**right side**). A: vascular structure; B: intense red area (lava-like); C: faint pink or pinkish area; D: blue area; E: cortical surface.

**Figure 2:**
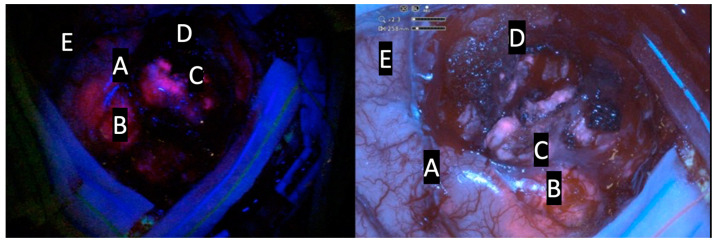
Comparative intraoperative pictures of patient N.8; kinevoi900 (**left side**); Olympus ORBEYE exoscope (**right side**). A: vascular structure; B: intense red area (lava-like); C: faint pink or pinkish area; D: blue area; E: cortical surface.

**Figure 3:**
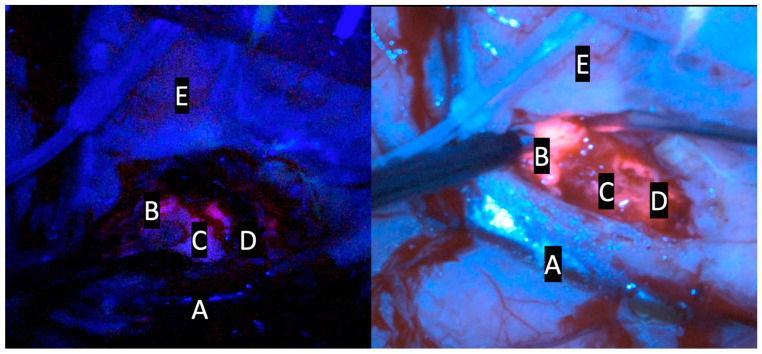
Comparative intraoperative pictures of patient N.2; kinevoi900 (**left side**); Olympus ORBEYE exoscope (**right side**). A: vascular structure; B: intense red area (lava-like); C: faint pink or pinkish area; D: blue area; E: cortical surface.

The introduction of the exoscope in the modern era provided neurosurgeons with a digital camera system able to deliver intense light and magnification to the deepest areas of the surgical field, allowing surgeon to see, through a 3D 4K monitor, critical neural and vascular structures as well as tissue differentiation with high magnification. Several studies demonstrated that the exoscope could be considered a safe alternative compared to the OM thanks to the better ergonomic posture of surgeons during surgical procedures and thanks to the possibility to improve the operational team’s involvement. Furthermore, it has been demonstrated that the quality of images and the 3D 4K screen has been increasingly improved in recent years, giving high magnification and an enhanced image quality of the surgical field while also being more ergonomically favorable. Drawback remains about the slight difference in depth perception in exoscopic versus microscopic views [11,13]. Several studies have shown that fluorescence-guided tumor resection increases the possibility of achieving the GTR [16,17]. The possibility of applying a blue filter to the HGG 5-ALA-guided surgery in the exoscopic magnified surgical field is a further advantage. The results of this study demonstrate the ability of the ORBEYE exoscope to accurately identify tissue containing tumor cells compared to a traditional microscope, similarly to prior studies using 5-ALA with a standard operative microscope [7,9,18]. Recent reviews have underlined the feasibility of 5-ALA-guided surgery with an exoscopic guide: Vogelbaum et al. demonstrated that the visualization of 5-ALA-induced tumor fluorescence was associated with a positive predictive value as well as other 5-ALA visualization technologies; furthermore, the authors reported that this digital system allowed for an excellent visualization of both the fluorescence and the anatomy at the same time, which distinguishes this technology from conventional surgical microscopes (Figure 1, Figure 2 and Figure 3) [19].

These results are in line with our findings. Papers were published regarding pediatric neurosurgery applications, with good reports in determining the extent of resection [20]. Ikeda et al., reported a comparative study analyzing the technical characteristics of fluorescence-guided surgery with 5-aminolevulinic acid and an excitation light source with Olympus ORBEYE and a traditional microscope: the authors demonstrated that nonfluorescent normal structures without red fluorescence were well recognized under the exoscope; the energy of the 405 nm wavelength in blue light was significantly higher in the ORBEYE exoscope than that in the traditional microscope, especially in the short focal length, thus giving the exoscope a good visibility advantage due to blue light energy compared to a traditional microscope [21]. In our experience, there were no major differences in adapting from a traditional microscope to an exoscope in detecting fluorescence induced by 5-ALA. The major difference was found in the intensity of the fluorescence in the two groups, the brightness of the surgical field and the detailing of the anatomy while using the blue filter. In our opinion, a combination of these two technologies may lead to improved levels of the extent of resection, the control of the surgical field and complication rates.

## 5. Conclusions

This preliminary study demonstrates that the combination of fluorescence induced by 5-aminolevulinic acid and the exoscope system is possible. These two technologies allow a surgeon to perform fully collaborative surgeries, to add ergonomics to the procedure and to have a better visualization of the surgical blue-filtered field compared to the OM.

## Figures and Tables

**Table 1:** Clinical characteristics of cases included (10 consecutive patients who underwent surgery for the resection of an intra-axial lesion from February 2022 to February 2023 (WHO: World Health Organization; M: male; F: female; GTR: gross total resection, STR: subtotal resection).

Pt	WHO, 2021	Age	Gender	Location	Grade of Resection
1	Glioblastoma, 4	67	M	Right frontal	GTR
2	Glioblastoma, 4	80	F	Right frontal	GTR
3	Diffuse glioma, 4	66	M	Right temporal	GTR
4	Diffuse glioma, 4	39	M	Right fronto-parietal	GTR
5	Glioblastoma, 4	42	M	Right occipital	GTR
6	Glioblastoma, 4	61	M	Right insular	STR
7	Glioblastoma, 4	58	F	Right fronto-temporal	GTR
8	Glioblastoma, 4	64	M	Left frontal	GTR
9	Glioblastoma, 4	70	M	Left parietal	GTR
10	Glioblastoma, 4	74	F	Left frontal	GTR

**Table 2:** Fluorescence emission classification.

Intensity of Fluorescence	Type of Tissue
Intense red or lava-like	Tumor
Faint pink or pinkish	Infiltrating tumor cells
Blue	Normal tissue

**Table 3:** Subjective assessment of the surgeons on a scale of 1–5 (OE group; OM group) of the characteristics examined: fluorescence intensity, brightness of the surgical field, detailing of the anatomy with a blue filter.

	Fluorescence Intensity (OE) (IR 4.5–5)	Brightness of the Surgical Field (OE) (IR 4.9–5)	Detailing of the Anatomy with a Blue Filter on OE (IR 4–5)
1	5	5	4.5
2	4.5	5	5
3	4	5	4.5
4	4.5	4.5	4
5	5	5	4.5
6	4	4.5	5
7	5	5	4
8	5	5	4
9	5	5	5
10	5	5	4.5
mean	4.7	4.9	4.5
	Fluorescence intensity (OM) (IR 4–4.125)	Brightness of the surgical field (OM) (IR 3.5–4)	Detailing of the anatomy with a blue filter on OM (IR 3–3.5)
1	4	3.5	3.5
2	4	4	3
3	3	4	3.5
4	4	3.5	3
5	4	4	3.5
6	4	3.5	3
7	4.5	4	3
8	4	4.5	2.5
9	4	4	3.5
10	4.5	3.5	3.5
mean	4.0	3.85	3.2

**Table 4:** Statistical analysis and *t*-test (SPSS statistics)*;* OM: operative microscope; OE: Olympus ORBEYE.

		Fluorescence Intensity, OM	Brightness of the Surgical Field, OE	Brightness of the Surgical Field, OM	Detailing of the Anatomy with a Blue Filter, OE	Detailing of the Anatomy with a Blue Filter, OM
N		10	10	10	10	10
Mean		4.0000	4.9000	3.8500	4.5000	3.2000
Median		4.0000	5.0000	4.0000	4.5000	3.2500
Std. Deviation		0.40825	0.21082	0.33747	0.40825	0.34960
Range		1.50	0.50	1.00	1.00	1.00
Minimum		3.00	4.50	3.50	4.00	2.50
Maximum		4.50	5.00	4.50	5.00	3.50
		One-Sided *p*	Two-Sided *p*		Lower	Upper
Fluorescence intensity, OE	35.250	<0.001	<0.001	4.70000	4.3984	5.0016
Fluorescence intensity, OM	30.984	<0.001	<0.001	4.00000	3.7080	4.2920
Brightness of the surgical field, OE	73.500	<0.001	<0.001	4.90000	4.7492	5.0508
Brightness of the surgical field, OM	36.076	<0.001	<0.001	3.85000	3.6086	4.0914
Detailing of the anatomy with the blue filter, OE	34.857	<0.001	<0.001	4.50000	4.2080	4.7920
Detailing of the anatomy with the blue filter, OM	28.945	<0.001	<0.001	3.20000	2.9499	3.4501

**Table 5:** Comparative quantitative analysis of fluorescence intensity values; OM: operative microscope; OE: Olympus ORBEYE.

	Red	Pink	Blue	Range
OM	230–255	170–210	80–140	80–255
OE	220–250	190–220	100–180	100–250

**Table 6:** Comparative quantitative analysis of brightness values (RGB: red, green, blue); OM: operative microscope; OE: Olympus ORBEYE.

		OM (Range 50–255)	OE (Range 80–255)
Red areas	Red	255	255
	Green	50	120
	Blue	50	90
Pink areas	Red	235	245
	Green	130	200
	Blue	200	110
Blue areas	Red	80	140
	Green	120	170
	Blue	255	210

## Data Availability

The authors confirm that the data supporting the findings of this study are available within the article.
